# Baptisia in Pneumonia

**Published:** 1887-04

**Authors:** G. W. Sherbino

**Affiliations:** Abilene, Texas


### BAPTISIA IN PNEUMONIA.

G. W. Sherbino, Abilene, Texas.

Mr. B. has been feeling poorly for two or three days. This morning he had a severe chill. I was called at five P. M. I find the following symptoms:

Aching in the muscles and bones; aching from head to foot; bed as hard as a board; "*pillow hard as a rock.*"

Coughing large quantities of rusty sputa; stitching pain in left chest; has to hold his chest with his hands ("Bry." Dros.); "*fan-like motion of alae nasi*" (Phos., "Lycopod."); *stupid and sleepy*, could hardly keep awake; total loss of appetite; very thirsty for large drinks of water; cough worse at night; several loose stools per day—very offensive; wanted to keep very still, as the least movement aggravated his cough and pain in the chest. (Bry.-alb.)

Temperature, first evening, 101°; pulse, 110; respirations, 32. The second evening, temperature, 103°; respirations, 40; pulse, 120. Third day, eight A. M., temperature, 99°; pulse, 88; respirations, 28.

Irritable from children making any noise or running across the floor. Mr. B. had pneumonia two years ago this winter. I attended him; he had as severe an attack as before.

Two years ago he was sick eight days; he had the same symptoms then as he had this time.

His brother was taken two days sooner with pneumonia; attended by a "regular;" he did not suffer from any neglect as regards medical attendance or nursing; he had all that. The last twenty-four hours of his life he took one quart of whisky to keep his heart acting, and he was seen by the pastor, who pronounced him drunk, as he had the singultus.

He died on the seventh day. My patient took nothing but fresh milk and pure cold water, and was convalescent on the third day; he only got one remedy in the minimum dose. I had a great many cases of pneumonia to treat last winter. I got them early in the disease, and none of them was sick longer than eight days, and Baptisia was indicated in the early stage, and the most of them were cured without the help of any other remedy. I gave it until the existing symptoms were all gone, and found in most all of my cases the pneumonia was gone, too.

There is one symptom this man had after the fever and the patient convalescent. When coughing he had a terrific pain in the left shoulder; it seemed as though his shoulder would fly to pieces, and his wife would have to hold his shoulder with her hand by grasping over the top with hand and fingers extending down and pinch as hard as she could. This I thought a peculiar symptom. I obtained other symptoms. The pain was brought on by a fit of coughing, and he had to get out of bed and swing his arm back and forth as fast as he could, as he said that was the only thing that would relieve it. Rubbing afforded some relief. I gave him one dose of Rhus-tox.$^{cm}$, and he was cured; needed no more medicine of any kind. For the pneumonia I gave Baptisia$^{dmm}$, Swan's. I had just received this graft from Dr. Swan the morning that Mr. B. was taken sick, and I was anxious to try this potency, and I had the satisfaction of testing the virtue of this wonderful remedy, too often overlooked and neglected, as I intended to cast it overboard if it made any failures, and I am glad to know that the higher we go the quicker we cure and the less medicine it takes.

May the Lord ever bless the dear doctors who have given us the high potencies and the law of similars.

